# Selective RNA Labeling by RNA-Compatible Type II Restriction Endonuclease and RNA-Extending DNA Polymerase

**DOI:** 10.3390/life12101674

**Published:** 2022-10-21

**Authors:** Hyesung Jo, Jiyun Beon, Seung Soo Oh

**Affiliations:** Department of Materials Science and Engineering, Pohang University of Science Technology (POSTECH), Pohang 37673, Korea

**Keywords:** nucleic acids, RNA cleavage, RNA modification, modified nucleotides, mRNA detection

## Abstract

RNAs not only offer valuable information regarding our bodies but also regulate cellular functions, allowing for their specific manipulations to be extensively explored for many different biological and clinical applications. In particular, rather than temporary hybridization, permanent labeling is often required to introduce functional tags to target RNAs; however, direct RNA labeling has been revealed to be challenging, as native RNAs possess unmodifiable chemical moieties or indefinable dummy sequences at the ends of their strands. In this work, we demonstrate the combinatorial use of RNA-compatible restriction endonucleases (REs) and RNA-extending polymerases for sequence-specific RNA cleavage and subsequent RNA functionalization. Upon the introduction of complementary DNAs to target RNAs, Type II REs, such as AvrII and AvaII, could precisely cut the recognition site in the RNA-DNA heteroduplexes with exceptionally high efficiency. Subsequently, the 3′ ends of the cleaved RNAs were selectively and effectively modified when Therminator DNA polymerase template-dependently extended the RNA primers with a variety of modified nucleotides. Based on this two-step RNA labeling, only the target RNA could be chemically labeled with the desired moieties, such as bioconjugation tags or fluorophores, even in a mixture of various RNAs, demonstrating the potential for efficient and direct RNA modifications.

## 1. Introduction

Due to the large variety of sequences, ribonucleic acids (RNAs) play diverse biological roles in all cell-based lives and even RNA viruses [1]. Even though messenger RNAs (mRNAs) constitute only ~4% of the total cellular RNAs, more than 300,000 mRNAs exist in a human cell. As the majority of transcriptomes, various non-coding RNAs (e.g., ribosomal RNAs, transfer RNAs, microRNAs, small interfering RNAs, small nucleolar RNAs, extracellular RNAs, and piwi-interacting RNAs) are also found in the cell, as they are involved in a wide range of cellular functions, such as gene regulation, silencing, and defining DNA methylation patterns [2,3]. Since distinguishing a target RNA from others is crucial for obtaining valuable information about the current state of the cell, various selective RNA manipulation techniques have been developed [4]. In particular, based on the Watson–Crick base pairing, chemically modified oligos have been rationally designed to complement targeted RNAs for many biological and clinical applications [5,6,7]. For example, when a mixture of RNAs is size-separated through gel electrophoresis, synthetic oligos labeled with radioactive, fluorescent, or chemical tags can be introduced to selectively visualize the complementary target RNAs [8,9]. In addition to the northern blot, direct RNA detection techniques involving molecular beacons, hairpin-shaped oligos with a fluorophore and quencher pair [10,11], and oligos modified with isolation tags (e.g., biotins, 2, 4-dinitrophenyl, and digoxigenin) can be used to separate specific RNAs from cells and tissue lysates [12].

Despite the simplicity and versatility, relying only on hybridization with target RNAs has several drawbacks. For instance, when complex and long RNAs are hybridization targets, short complementary oligos often suffer from off-targeting due to the frequent occurrence of mismatches or wobble pairs [13]. Moreover, to detect low-concentration RNAs, recognizable signals should be further amplified. Consequently, a typical 1:1 hybridization adduct frequently needs complex modifications of complementary oligos, such as the conjugation of multiple dyes or enzymes that react with chromogenic substrates [14]. Besides, the hybridization-based RNA recognition strongly depends on transient hydrogen bonds; therefore, under certain conditions (e.g., high temperature, low salt concentrations, and high organic solvent compositions) the preformed duplexes can easily be denatured [15]. 

Although the direct labeling of desired RNAs can be considered an attractive alternative, it is considered challenging as native RNAs have unmodifiable chemical moieties or indefinable dummy sequences at the ends of the strands [16,17,18,19]. For instance, 5′-capped mRNAs exhibit different lengths of sequence-independent poly A tails at 3′ ends, and the most abundant rRNAs possess unconserved terminal sequences, complicating terminal modifications of typical nucleic acids [20]. Hence, directly modifying target RNAs is still an unmet need for transcriptome- and cell-wide studies and applications.

**Figure 1:**
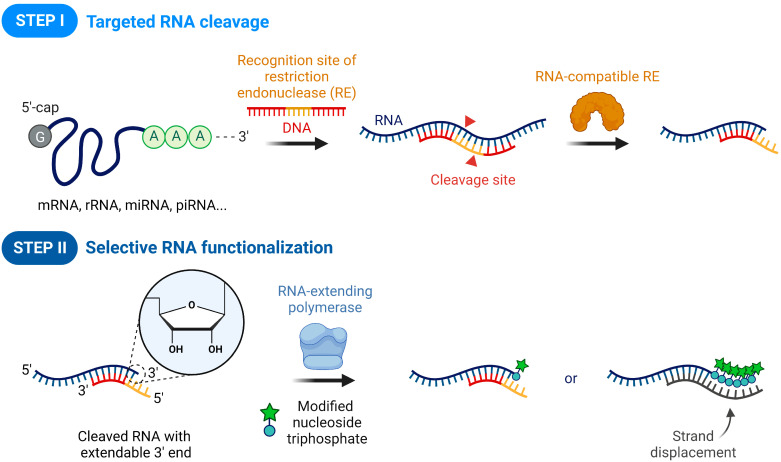
Schematic illustration of sequence-specific RNA functionalization using two consecutive steps, restriction endonuclease-based RNA cleavage and RNA-compatible polymerase-based terminal modification. STEP I: When a short synthetic DNA with a recognition site of restriction endonuclease (RE) hybridizes to a target RNA, the RNA-compatible Type II RE can recognize the RNA-DNA heteroduplex and subsequently cleave the designated site of both RNA and DNA, leaving a hybrid sticky end. STEP II: The resulting RNA fragment has a 3′-hydroxyl group, allowing further nucleotide insertion by RNA-extending polymerase as the 5′-overhang of the cleaved DNA serves as a DNA template. Upon the addition of modified nucleoside triphosphates, direct RNA functionalization can be achieved with the modified nucleotide-tolerating polymerase. Alternatively, a new DNA template can be introduced by strand displacement, allowing for the incorporation of multiple modified nucleotides at the 3′ end of the target RNA.

To selectively label RNAs, irrespective of modifications and sequence variations at both ends, fully sequence-defined mid-domains of RNAs with no complex folding structures are attractive targets for manipulation. In this work, we explore this possibility demonstrating a novel RNA modification method that combines RNA-DNA heteroduplex-mediated site-specific cleavage of Type II restriction endonucleases (REs) and modified nucleotide-assisted terminal modification of RNA-extending DNA polymerases (Figure 1).

There are four types of REs that recognize and cleave specific restriction sites. These sites typically reside in DNA-DNA homoduplexes, but hundreds of certain Type II REs, including AvaII and AvrII, have been revealed to sequence-specifically hydrolyze RNA-DNA heteroduplexes as well [21]. Due to the variety of possible restriction sites, the specific mid-domain of target RNAs can be targeted by bind to complementary DNA (cDNA) strands with the desired RE recognition sequence. Hence, in the presence of rationally designed cDNAs and RNA-compatible REs, the restriction sites of the target RNAs can be efficiently cleaved to leave specific sticky ends (Figure 1, STEP I). Whereas RNA cleavage mediated by chemicals, ribonucleases, ribozymes, and DNAzymes often produces a 2′,3′-cyclic phosphate as the 3′-terminal form of RNAs [22,23,24,25,26], in the case of the Type II REs cleavage, the cyclization product is not formed, allowing for further modifications, such as ligation and nucleotide extension [27]. Accordingly, the introduction of polymerases capable of extending RNA primers with modified nucleoside triphosphates, allows for 3′ end modifications of the cleaved RNAs on the residual DNA templates of the sticky ends or a new template introduced by strand displacement (STEP 2). We confirmed that Therminator DNA polymerase was optimal for the direct labeling of various functionalized moieties to the target RNAs. In addition, the possibility of fluorescent signal amplification for the sensitive detection of specific RNAs in a mixture of various RNAs was demonstrated.

## 2. Materials and Methods

### 2.1. Materials

Unless otherwise stated, all reagents used were purchased from Sigma-Aldrich (St. Louis, MO, USA) and Thermo Fisher Scientific (Waltham, MA, USA). All the DNAs were synthesized by Integrated DNA Technologies, Inc. (IDT, Coralville, IA, USA), and the sequence information is attached to the Supplementary Materials (Table S1). Restriction enzymes, AvrII and AvaII, and polymerases, Therminator DNA polymerase, Bsu DNA polymerase, Klenow fragment, and AMV reverse transcriptase, were purchased from New England Biolabs (Ipswich, MA, USA). Triphosphate forms of modified nucleosides were purchased from TriLink BioTechnologies (San Diego, CA, USA).

### 2.2. In Vitro RNA Transcription

To generate RNAs, in vitro transcription reactions were performed with 1 μg of linearized DNAs for 12 h at 37 °C. The reaction volume was 100 μL containing 100 units of T7 RNA polymerase (Bioneer, Daejeon, Republic of Korea), ATP (10 mM), GTP (10 mM), CTP (10 mM), UTP (10 mM), MgCl_2_ (75 mM), Dithiothreitol (40 mM), and T7 RNA polymerase buffer (Bioneer, Daejeon, Republic of Korea) containing 200 mM Tris-HCl, 30 mM MgCl_2_, 10 mM spermidine, and then DNase I treatment was conducted by using 5 units of DNase I for 15 min at room temperature. The transcribed RNA was electrophoresed on 10% denaturing polyacrylamide gel. Next, as visualized by UV shadowing, the transcript-including gel was cut out with a blade. To recover the transcripts, the collected gel was crushed in a clean tube, and the transcripts were eluted for 12 h in nuclease-free water. The crushed gel was centrifuged for 5 min at 10,000 *g*, and then the supernatant was collected in a tube, followed by ethanol precipitation. The purity and quantity of transcribed RNAs were measured by a spectrophotometer (DS-11+ Spectrophotometer, DeNovix, Wilmington, DE, USA).

### 2.3. Restriction Endonuclease Activity Assay Using RNA-DNA Heteroduplex

For the cleavage reaction by AvrII, a mixture containing RNA (10 pmol), DNA (20 pmol), 10× rCutSmart buffer (1 μL) and 25 units of AvrII with a total volume of 10 μL was incubated at 37 °C for 3 h, and heated at 80 °C for 20 min. For the cleavage reaction using AvaII, water was added to the solution containing RNA (10 pmol), DNA (20 pmol), 10× rCutSmart buffer (2 μL), and 100 units of AvaII up to 20 μL. The reaction was taken at 37 °C for 3 h, and, for the inactivation, the mixture was heated at 80 °C for 20 min. Before running gel electrophoresis, the sample purification was performed using ethanol precipitation by adding 3 M acetate buffer (2 μL, pH = 5.5) cold ethanol (66 μL) and glycogen (20 mg/mL, 1.5 μL). After placing in a freezer at −80 °C for 40 min, the nucleic acid precipitate was collected by centrifugation for 30 min (20,000 g).

### 2.4. Modified Nucleotide Insertion Using Polymerases

Modified nucleotide insertion using different polymerases was conducted in a 20 μL solution with 6-carboxyfluorescein (FAM)-labeled RNA (20 pmol), DNA template (40 pmol), modified nucleoside triphosphate (2 nmol), 2 μL of 10× buffer provided with each enzyme (NEB, Ipswich, MA, USA), and 2/2.5/2.5/5 units of Therminator polymerase/Bsu polymerase/Klenow fragment/AMV reverse transcriptase, respectively. The mixture with Thermiantor polymerase was incubated for 2 h at 55 °C, while those using other enzymes were incubated at 37 °C. The sequence information used in this experiment is provided in the Supplementary Materials (Table S1).

### 2.5. Denaturing Gel Electrophoresis

Denaturing urea polyacrylamide gel electrophoresis was used for the evaluation of oligonucleotides. To prepare 10% polyacrylamide/7.5 M urea gel, UreaGel diluent (T&I biotechnology, Hebei, China) was added to T&I UreaGel 29:1 concentrate (25%, 12 mL), UreaGel buffer (10×, 3 mL), and tetramethylethylenediamine to make a total volume of 30 mL. Then, freshly prepared ammonium persulfate (10%, 200 μL) was added, and the mixture was gently shaken before pouring onto the cassette of 25 × 21 cm (W × H). After the samples were loaded on the gel, the gel was run at 30 Watts for 80 min using PowerPac High-Voltage Power Supply (Biorad, Hercules, CA, USA).

## 3. Results and Discussion

### 3.1. Site-Specific Cleavage of RNA-DNA Heteroduplexes

**Figure 2:**
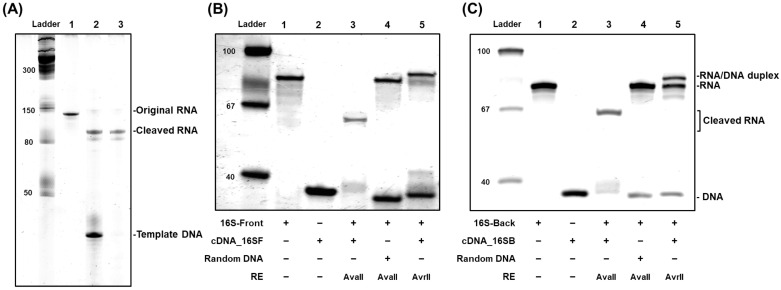
Successful sequence-specific cleavage of in-vitro-transcribed RNAs by RNA-compatible REs. (**A**) AvrII-mediated RNA-DNA heteroduplex cleavage. Lane 1. CR (108 nt-long RNA); 2. CR + cCR (40 nt-long cDNA of CR) + AvrII; 3. CR + cCR + AvrII and DNase I treatment. (**B**) Site-selective cleavage of 16S rRNA front fragment by AvaII. Lane 1. 16S-Front (80 nt-long 16S rRNA_877-956_); 2. cDNA of 16S-Front; 3. 16S-Front + cDNA + AvaII; 4. 16S-Front + scrambled DNA + AvaII; 5. 16S-Front + cDNA + AvrII. (**C**) Site-selective cleavage of 16S rRNA back fragment by AvaII. 1. 16S-Back (80 nt-long 16S rRNA_926-1005_); 2. cDNA of 16S- Back; 3. 16S- Back + cDNA + AvaII; 4. 16S- Back + scrambled DNA + AvaII; 5. 16S- Back t + cDNA + AvrII. Ladder: molecular size markers in base pairs. All the gels were stained by SYBR Gold.

When a long, transcribed RNA was partially hybridized with a short synthetic cDNA, Type II restriction endonucleases successfully recognized the desired restriction cleavage sites in the RNA-DNA hybrid duplex and subsequently cleaved both DNA and RNA with exceptionally high efficiency (Figure 2A). For validation, a certain RNA (CR, 108 nt) was in vitro transcribed by T7 RNA polymerase (Figure 2A, lane 1), and the rationally designed cDNA (cCR, 40 nt) was next introduced to induce the formation of RNA-DNA heteroduplex, of which mid-domain contains 5′-CCUAGG-3′/5′-CCTAGG-3′ for the restriction site of AvrII (see Methods). Upon incubation with the AvrII REs at 37 °C, both DNAs and RNAs were readily cleaved at the desired restriction site to generate the sticky end, 5′-C-3/5′-CTAGG-3′. In the denaturing gel electrophoresis, we observed the appearance of the cleaved RNA fragments (lane 2, 83 nt) along with the DNA fragments cut together by the AvrII REs (21 nt). We note that, as a result of the cleavage by the REs, the band of the original transcribed RNAs (108 nt) almost disappeared, demonstrating that the cutting efficiency of REs would be close to 100%. To further confirm the identity of the band, we performed DNase I treatment (lane 3), observing that the upper band was intact, but the lower one vanished. This result indicated that the mid-domain of the transcribed RNAs was indeed cleaved by the assistance of the cDNAs and the REs.

Depending on the RE, different sites of long RNAs could be cleaved selectively and efficiently (Figure 2B,C). As 16S rRNA of Staphylococcus aureus, one of the well-known pathogens to cause infectious diseases in humans, is identified to contain several restriction sites of AvaII, 5′-GG(A/U)CC-3′, we prepared two different fragments, 16S-Front (16S rRNA_877-956_, 80 nt, lane 1 in Figure 2B) and 16S-Back (16S rRNA_926-1005_, 80 nt, lane 1 in Figure 2C) for the investigation of site-specific cleavage by AvaII REs. When appropriate cDNAs (cDNA_16SF and cDNA_16SB, lane 2 in Figure 2B,C, respectively) were introduced with AvaII, the formed RNA–DNA heteroduplexes showed successful cleavage of RNAs with almost 100% efficiency (lane 3 in Figure 2B,C). For the successful RNA cleavage, the restriction site within the heteroduplex was the only important factor. Even though the sequences of the 16S-Front and the 16S-Back were chosen to include 5′-GGACC’-3 near the 5′ and 3′ end, respectively, with different flanking sequences, both RNAs exhibited roughly 100% yields in cleavage by the correct REs. However, when we introduced arbitrary DNAs with the same lengths as the original cDNAs, the bands of the original RNAs were intact (lanes 4 in Figure 2B,C), revealing that, without the correct hybridization cleavage of target RNAs did not take place. Moreover, REs that recognize other restriction sites did not cause RNA cleavage, as demonstrated by the replacement of AvaII with AvrII (lane 5 in Figure 2B,C). From all these observations, we conclude that our cDNA-guided RNA cleavage by REs offers an accurate way to cause a sticky end at the desired position of RNA through two-step sequence confirmation: the sequence-specific hybridization of the target RNA with the cDNA and the restriction site-specific cleavage of RNA-DNA heteroduplexes.

### 3.2. Sequence-Specific RNA Labeling Based on RNA-DNA Hybrid Sticky Ends

**Figure 3:**
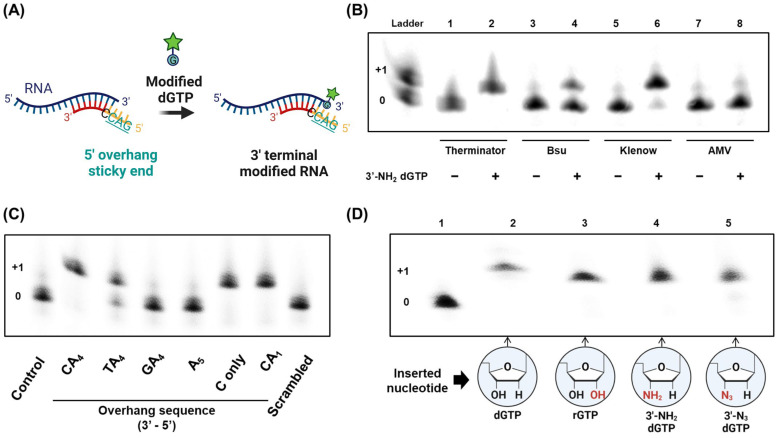
Modified nucleotide insertion by RNA-compatible polymerase based on RNA-DNA hybrid sticky ends. (**A**) Schematic representation of selective RNA labeling using 5′ DNA overhangs. AvaII-induced restriction site-specific cleavage leads to the generation of RNA-DNA duplex fragments with 5′ DNA overhangs of 5′-GAC-3′, which serve as a DNA template for 3′ extension of RNA primers by modified nucleotide-tolerating polymerases. Upon the addition of modified dGTPs, one guanosine with a desired functional group can be selectively added to the complementary site of cytosine located in the 5′ DNA overhang. (**B**) RNA labeling using various RNA-compatible polymerases and dGTP with 3′-NH_2_. (**C**) Fidelity of Therminator in nucleotide addition. The sequence of 5′ DNA overhangs was varied to confirm the high accuracy and efficiency in selectively inserting a modified nucleotide, 3′-amino-2′3′-dideoxyguanosine monophosphate. (**D**) Efficient RNA labeling with diverse functional groups based on the use of different modified nucleoside triphosphates. dGTP, rGTP, dGTP with 3′-NH_2_, and dGTP with 3′-N_3_ were used to modify the 3′ end of target RNAs by Therminator. All the gels were stained by SYBR Gold.

Availability of RNA-DNA hybrid sticky ends can be used for enzymatic 3′ end modification of RNAs. For example, after the AvaII-based restriction site cleavage, the 5′ DNA overhang, 5′-GAC’-3′, can serve as a DNA template for the polymerase to further extend the 3′ end of the cleaved RNA. In the presence of modified 2′-deoxyguanosine 5′-triphosphates (dGTPs), one modified G would be added for selective and quantitative labeling of the target RNAs (Figure 3A). To this end, it was necessary to determine an optimal polymerase capable of utilizing a triphosphate form of modified nucleosides in extending RNA primers even on the 5′ overhang of DNA templates. For this purpose, we chose three different DNA polymerases, Therminator, Bsu, and the Klenow fragment, and one reverse transcriptase (RT) from avian myeloblastosis virus (AMV), as all of them have been known to extend the 3′ end of RNAs. Then, we examined the labeling performance of each polymerase using 14nt-long RNA fragments and 3′-NH_2_-modified dGTPs with cDNAs with 5′-GAC-3′ overhangs (Figure 3B). Therminator exhibited the best extension efficiency among the chosen enzymes, allowing for all the RNAs (Figure 3B, lane 1) to be fully labeled with deoxyguanosine with 3′-NH_2_ (lane2). Comparably, Bsu polymerase and the Klenow fragment showed poor activities in RNA labeling with an extension rate of 37% (lane 4) and 88.6% (lane 6), respectively. No extension was found when AMV RT was used on the DNA template (lane 8), but it is worth noting that more than half of the RNA fragments were elongated on an RNA template (Figure S1).

For accurate RNA labeling, the fidelity of the polymerase must be taken into consideration. By varying the sequence of 5′ DNA overhangs, we investigated the accuracy in the incorporation of modified nucleotides to the 3′ end of RNAs (Figure 3C). Due to the highest extension rate of modified nucleotides based on the RNA-DNA hybrid sticky ends, Therminator polymerase was used for the fidelity experiments. When 5′-AAAAC-3′ overhangs served as an extension template, one 3′-amino-2′3′-dideoxyguanosine monophosphate was successfully added to the 3′ end of 14 nt-long RNAs (FAM_R) based on Watson–Crick base pairing (Figure 3C, CA_4_). The replacement of C with T in the DNA overhang caused some degree of G insertion by potential wobble base pairs (TA_4_), while overhangs with G and A induced no elongation of RNAs by Therminator at all (GA_4_, and A_5_, respectively). Even though the yield of nucleotide addition close to the 5′ end of DNA template is reduced, it was confirmed that only one G was inserted with high fidelity and efficiency on either 5′-C-3′ or 5′-AC-3′ overhangs (C only and CA_1_, respectively). No RNA elongation was found upon the introduction of scrambled DNA templates (Scrambled), once again demonstrating the importance of correctly hybridized RNA-DNA hybrid sticky ends in RNA labeling. We note that when the same experiments were conducted with the Klenow fragment, the rate of RNA elongation was much lower than that with Therminator, and the nucleotide incorporation was highly influenced by the presence of the adjacent bases (Figure S2).

A variety of modified nucleotides could be inserted into the 3′ end of RNAs by the use of Therminator polymerase. As representative examples, we prepared dGTP, rGTP, dGTP with 3′-NH_2_ and 3′-N_3_ to examine the RNA elongation efficiency of Therminator and the Klenow fragment on the DNA template that contains 5′-GAC-3′ overhangs (Figure 3D and Figure S3). In addition to the triphosphate forms of the deoxynucleotide and the ribonucleotide (dGTP and rGTP, respectively), which are the basic building blocks of DNAs and RNAs, 3′-amino- and 3′-azido-2′3′-dideoxyguanosine triphosphates were examined because the amine and azide groups are widely used for 1-ethyl-3-(3-dimethylaminopropyl)carbodiimide(EDC)/N-hydroxysuccinimide(NHS) coupling, one the most common bioconjugate chemistries, and the highly efficient click reaction, respectively. In denaturing gel electrophoresis, Therminator showed the excellent insertion of all chosen nucleotides, regardless of functional moieties at the 3′ end (Figure 3D, lane 2–5), confirming that this DNA polymerase was widely applicable for the selective extension of RNA primers with modified nucleotides on DNA templates. We also note that the Therminator DNA polymerase has been used for the introduction of various nucleotides on both DNA and RNA substrates using rNTPs, functionalized dNTPs, and even xeno nucleic acids (XNAs) [28]. However, the Klenow fragment only exhibited full insertion in the presence of dGTPs (Figure S3). Even though the use of dGTP with 3′-NH_2_ caused some degree of nucleotide addition by the Klenow fragment, the use of rGTP and dGTP with 3′-N_3_ resulted in almost no incorporation, demonstrating the superiority of Therminator in RNA labeling.

### 3.3. Selective Labeling of Target RNA and RNA Functionalization

**Figure 4:**
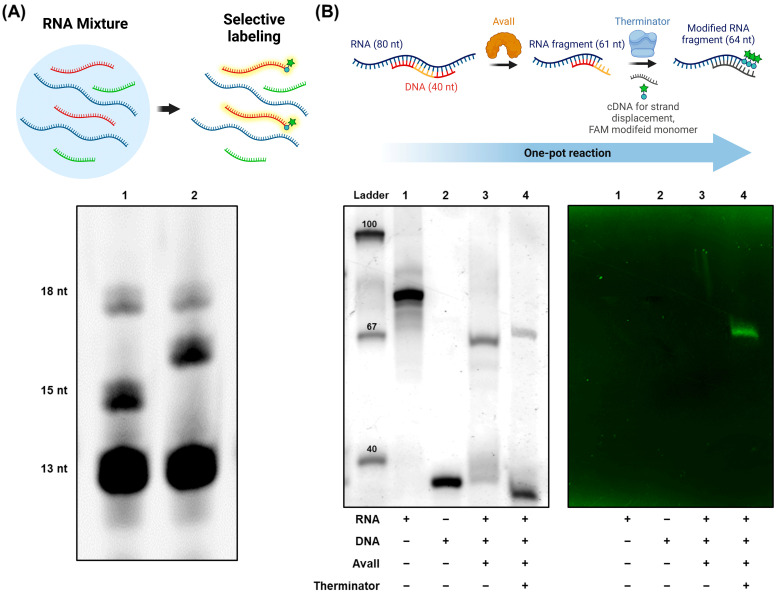
Selective labeling of target RNAs in one pot. (**A**) Sequence-specific targeting in RNA labeling by Therminator. Lane 1. RNA13 + RNA15 + RNA18; 2. RNA13 + RNA15 + RNA18 + cDNA of RNA15 + Therminator + dTTP with 3′NH_2_. The gel was stained by SYBR Gold. (**B**) RNA cleavage and terminal modification via the one-pot reaction. Lane 1. 16S-Front; 2. cDNA of 16S-Front; 3. 16S-Front + + cDNA + AvaII; 4. 16S-Front + + cDNA + AvaII + Terminator + FAM-labeled nucleoside triphosphate. (left: the gel stained by SYBR Gold, right: fluorescence at 526 nm with no staining). Ladder: molecular size markers in bp.

Based on the sequence specificity of cDNA hybridization and RNA cleavage, we further investigated the feasibility of RNA targeting in labeling using a mixture of various RNAs and a target-specific cDNA (Figure 4A). In a solution containing an equivalent amount of three RNAs of 13, 15, and 18 nt lengths (RNA13, RNA15, and RNA18, respectively, see lane 1), the cDNA designed to hybridize to the RNA15 with 5′-ACC-3′ overhangs at the 5′ ends, was added together with Therminator and dTTP with 3′-NH_2_. As a result, one 3′-amino-2′3′ dideoxythymidine monophosphate was successfully added to the targeted RNA15 with almost 100% efficiency (lane 2). This observation clearly demonstrates the potential of our methodology in multiplexed and biorthogonal RNA functionalization wit hthe use of well-designed multiple cDNAs with varied overhangs.

Finally, we examined the possibility of a one-pot procedure in which the RNA cleavage by restriction site-specific RE and RNA functionalization by DNA template-dependent polymerase steps are performed without purification in between (Figure 4B). When the 16S-Front RNA (lane 1) was targeted by its cDNA (lane 2), the presence of AvaII induced the sequence-specific RNA cleavage as evidenced by the appearance of 61 nt-long cleaved RNAs (lane 3). Once cleaved, the RNAs could be newly hybridized with another cDNAs with 5′-AAA-3′ overhangs at the 5′ end (cDNA-AAA) by strand replacement. Subsequently, in the presence of Therminator and FAM-labeled uridine triphosphate, multiple fluorescent nucleotides were added to the 3′ end of the cleaved RNAs (lane 4). As there were three available spots for the FAM-labeled nucleotides to be extended by Therminator, the target RNAs labeled with multiple fluorophores emitted strong fluorescent signals even in the absence of staining. In sum, through the combination of RNA-compatible RE and polymerase, target RNAs could be readily modified even with multiple functional groups via a simple DNA template design allowing for fluorescent detection of the target RNA.

## 4. Conclusions

In this study, we investigated whether simple direct modifications on complex RNAs are feasible, and experimentally validated that the successive application of RNA-compatible REs and RNA-extendable DNA polymerases allows for sequence-specific RNA functionalization with assistance from rationally designed cDNAs. Our RNA labeling technique is readily applicable for native RNAs that often possess unclear terminal domains and unmodifiable capping moieties. Due to the cDNA-guided site-specific cleavage of mid-domain RNAs, the target RNAs expose well-defined 3′ ends that permit direct chemical modifications with high efficiency. Among the polymerases that can catalyze template-directed DNA synthesis using RNA primers, we found that Therminator DNA polymerase has the highest potency for the insertion of various functional nucleotides. We also demonstrated that the Therminator-guided terminal labeling takes place selectively for the desired RNA when other RNAs are present. Moreover, by changing the cDNA through strand displacement, we verified that several functional monomers can be added at the 3′ end of the cleaved RNA, revealing the potential of signal amplification. 

For the direct labeling of target RNAs, the two consecutive steps of RNA cleavage and enzymatic modification can be simply performed in one pot under mild conditions, but there are still several limitations. For instance, the target RNAs must contain the RE recognition site and the triphosphate form of monomers with desired functional groups must be present. However, more than 3000 REs with varying recognition sites are currently available and it is anticipated that the catalogue of RNA-compatible REs could be further expanded via the engineering of RE variants. Moreover, nucleoside triphosphates with a variety of functional groups, such as fluorescent dyes, quenchers, attachment moieties, and modifiable linkers, have already been developed [29], and the discovery of genetically modified enzymes capable of expanding the availability of noncanonical residues is ongoing with the advances in the field of nucleic acid engineering [30]. Given the availability of various RNA-processing enzymes, we envision that this unique RNA-labeling technique would serve as a versatile alternative to the conventional hybridization-based RNA manipulations and broaden the field of RNA engineering.

## Data Availability

Not applicable.

## Supplementary Materials

The following supporting information can be downloaded at: https://www.mdpi.com/article/10.3390/life12101674/s1, Figure S1: RNA primer extension using AMV RT and dGTP with 3′-NH_2_; Figure S2: Fidelity of nucleotide addition by Klenow fragment; Figure S3: RNA labeling by Klenow fragment; Table S1: Oligonucleotide sequence.

## References

[1] Zong D., Oberdoerffer P., Batista P.J., Nussenzweig A. (2020). RNA: A double-edged sword in genome maintenance. Nat. Rev. Genet..

[2] Statello L., Guo C.-J., Chen L.-L., Huarte M. (2021). Gene regulation by long non-coding RNAs and its biological functions. Nat. Rev. Mol. Cell Biol..

[3] Han M., Beon J., Lee J.Y., Oh S.S. (2021). Systematic combination of oligonucleotides and synthetic polymers for advanced therapeutic applications. Macromol. Res..

[4] Ouyang T., Liu Z., Han Z., Ge Q. (2019). MicroRNA Detection specificity: Recent advances and future perspective. Anal. Chem..

[5] Paillasson S., van de Corput M., Dirks R.W., Tanke H.J., Robert-Nicoud M., Ronot X. (1997). In situ hybridization in living cells: Detection of RNA molecules. Exp. Cell Res..

[6] Park G., Kang B., Park S.V., Lee D., Oh S.S. (2021). A unified computational view of DNA duplex, triplex, quadruplex and their donor-acceptor interactions. Nucleic Acids Res..

[7] Yoo H., Jo H., Oh S.S. (2020). Detection and beyond: Challenges and advances in aptamer-based biosensors. Mater. Adv..

[8] Sawai T., Uzuki M., Koji T. (2000). In situ hybridization for RNA: Radioactive DNA probe. Molecular Histochemical Techniques.

[9] Unger E.R. (1990). In situ hybridization: Principles and practice. Clin. Immunol. Newsl..

[10] Chen C., Ridzon D.A., Broomer A.J., Zhou Z., Lee D.H., Nguyen J.T., Barbisin M., Xu N.L., Mahuvakar V.R., Andersen M.R. (2005). Real-time quantification of microRNAs by stem-loop RT-PCR. Nucleic Acids Res..

[11] Tyagi S., Kramer F.R. (1996). Molecular beacons: Probes that fluoresce upon hybridization. Nat. Biotechnol..

[12] Ried T., Baldini A., Rand T.C., Ward D.C. (1992). Simultaneous visualization of seven different DNA probes by in situ hybridization using combinatorial fluorescence and digital imaging microscopy. Proc. Natl. Acad. Sci. USA.

[13] Arvey A., Hermann A., Hsia C.C., Ie E., Freund Y., McGinnis W. (2010). Minimizing off-target signals in RNA fluorescent in situ hybridization. Nucleic Acids Res..

[14] Yang H., Wanner I.B., Roper S.D., Chaudhari N. (1999). An optimized method for in situ hybridization with signal amplification that allows the detection of rare mRNAs. J. Histochem. Cytochem..

[15] Cui S., Yu J., Kühner F., Schulten K., Gaub H.E. (2007). Double-stranded DNA dissociates into single strands when dragged into a poor solvent. J. Am. Chem. Soc..

[16] Necsulea A., Soumillon M., Warnefors M., Liechti A., Daish T., Zeller U., Baker J.C., Grützner F., Kaessmann H. (2014). The evolution of lncRNA repertoires and expression patterns in tetrapods. Nature.

[17] Hezroni H., Koppstein D., Schwartz M.G., Avrutin A., Bartel D.P., Ulitsky I. (2015). Principles of long noncoding RNA evolution derived from direct comparison of transcriptomes in 17 Species. Cell Rep..

[18] Chen J., Shishkin A.A., Zhu X., Kadri S., Maza I., Guttman M., Hanna J.H., Regev A., Garber M. (2016). Evolutionary analysis across mammals reveals distinct classes of long non-coding RNAs. Genome Biol..

[19] Ruan X., Li P., Chen Y., Shi Y., Pirooznia M., Seifuddin F., Suemizu H., Ohnishi Y., Yoneda N., Nishiwaki M. (2020). In vivo functional analysis of non-conserved human lncRNAs associated with cardiometabolic traits. Nat. Commun..

[20] Clark D.P., Pazdernik N.J., McGehee M.R. (2019). Basic genetics. Molecular Biology.

[21] Murray I.A., Stickel S.K., Roberts R.J. (2010). Sequence-specific cleavage of RNA by type II restriction enzymes. Nucleic Acids Res..

[22] Breslow R. (1993). Kinetics and mechanism in RNA cleavage. Proc. Natl. Acad. Sci. USA.

[23] Shigematsu M., Kawamura T., Kirino Y. (2018). Generation of 2′,3′-cyclic phosphate-containing RNAs as a hidden layer of the transcriptome. Front. Genet..

[24] Liu H., Yu X., Chen Y., Zhang J., Wu B., Zheng L., Haruehanroengra P., Wang R., Li S., Lin J. (2017). Crystal structure of an RNA-cleaving DNAzyme. Nat. Commun..

[25] Yoo H., Lee J.Y., Park K.S., Oh S.S. (2022). Lead-start isothermal polymerase amplification controlled by DNAzymatic switches. Nanoscale.

[26] Park S.V., Yang J.-S., Jo H., Kang B., Oh S.S., Jung G.Y. (2019). Catalytic RNA, ribozyme, and its applications in synthetic biology. Biotechnol. Adv..

[27] Watanabe T., Watanabe T. (2018). Chapter 4—The Cell. Biophysical Basis of Physiology and Calcium Signaling Mechanism in Cardiac and Smooth Muscle.

[28] Gardner A.F., Jackson K.M., Boyle M.M., Buss J.A., Potapov V., Gehring A.M., Zatopek K.M., Corrêa I.R., Ong J.L., Jack W.E. (2019). Therminator DNA polymerase: Modified nucleotides and unnatural substrates. Front. Mol. Biosci..

[29] McKenzie L.K., El-Khoury R., Thorpe J.D., Damha M.J., Hollenstein M. (2021). Recent progress in non-native nucleic acid modifications. Chem. Soc. Rev..

[30] Coulther T.A., Stern H.R., Beuning P.J. (2019). Engineering polymerases for new functions. Trends Biotechnol..

