# Supplementary material for: Selective RNA Labeling by RNA-Compatible Type II Restriction Endonuclease and RNA-Extending DNA Polymerase
Source: Life (Basel). 2022 Oct 21;12(10):1674. doi: 10.3390/life12101674 (PMC9605241; doi:10.3390/life12101674)
Supplement: Supplementary file 1 [file life-12-01674-s001.zip › life-1974200-supplementary.pdf]

life

Supplementary Materils

Selective RNA Labeling by RNA-Compatible Type II Restriction Endonuclease and RNA-Extending DNA Polymerase

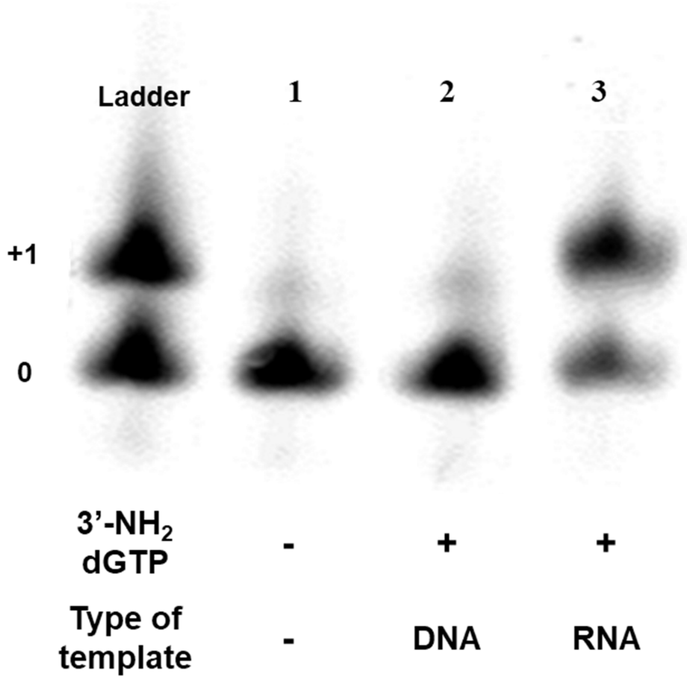

Figure S1. RNA primer extension using AMV RT and dGTP with 3′-NH_2. Almost no elongation was observed when a DNA template was used (lane 2); however, the use of RNA template allowed 64.98 % of modified nucleotide insertion to the 3′ end of the RNA (lane 3). The gel was stained by SYBR Gold.

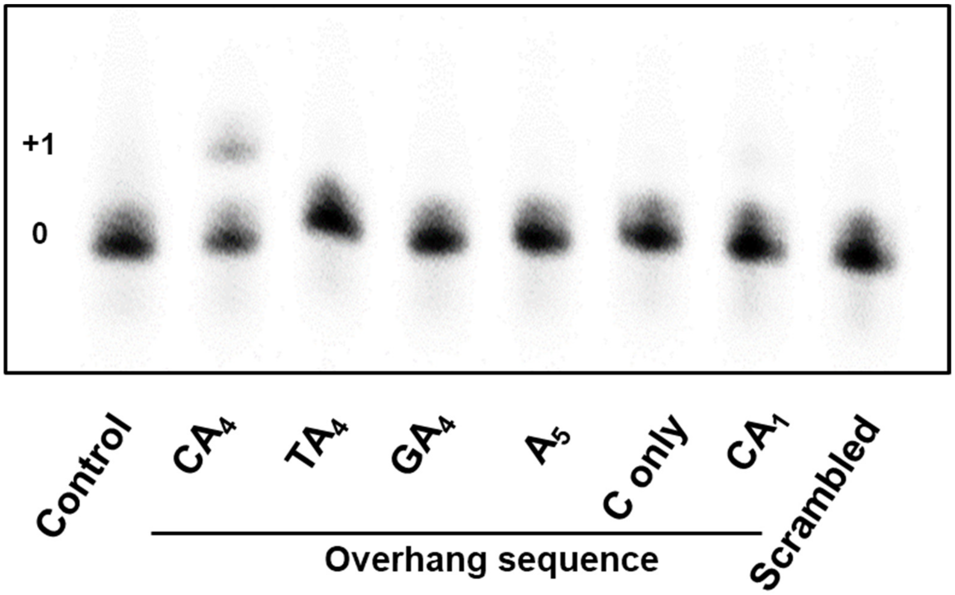

Figure S2. Fidelity of nucleotide addition by Klenow fragment. The sequence of 5′ DNA overhangs was varied to verify the accuracy and efficiency of modified nucleotide insertion. The gel was stained by SYBR Gold.

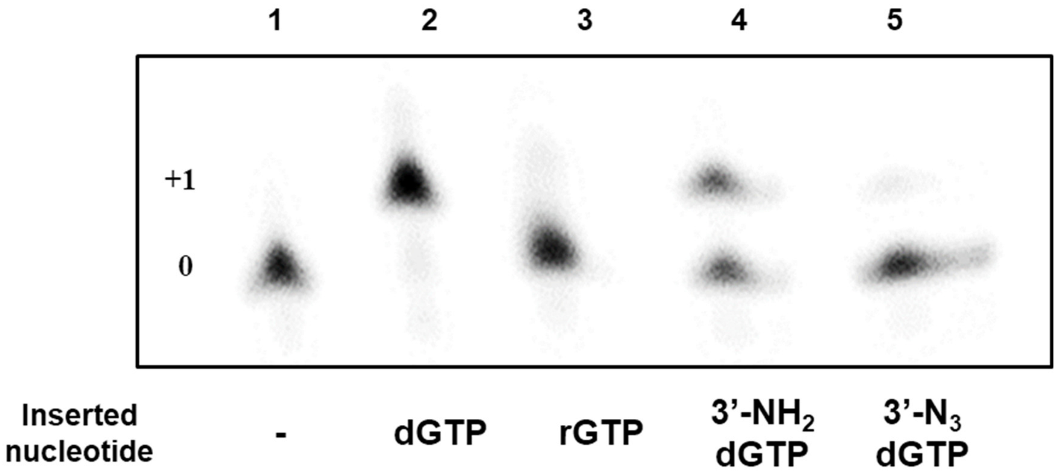

Figure S3. RNA labeling by Klenow fragment. Different modified nucleoside triphosphates, dGTP, rGTP, dGTP with 3′-NH_2, and dGTP with 3′-N_3, were used with the Klenow fragment for the insertion of modified nucleotide at 3′ end of the target RNA. The gel was stained by SYBR Gold.

Table S1. Oligonucleotide sequence. All recognition sites of restriction endonucleases are shown in red. All RNAs except for 5′ fluorescein-modified RNAs were transcribed via in vitro transcription.

Name	Type	Sequence
CR	RNA	5′ GGU CGU CAG UGC AUU GAG AAG GAG GAU AAA AUG CAC AUA GGU CGA AAG ACC UUA UAC AAG AAC UGU AUC ACC GGA GGG CGA CCC UAG GCU GUU AUU GCC GCU CUC AUC 3′
cCR	DNA	5′ GAT GAG AGC GGC AAT AAC AGC CTA GGG TCG CCC TCC GGT G 3′
16S-Front	RNA	5′ GCA TTA AGC ACT CCG CCT GGG GAG TAC GAC CGC AAG GTT GAA ACT CAA AGG AAT TGA CGG GGA CCC GCA CAA GCG GTG GA 3′

cDNA_16SF	DNA	5′ TCC ACC GCT TGT GCG GGT CCC CGT CAA TTC CTT TGA G 3′
16S-Back	RNA	5′ GGA AUU GAC GGG GAC CCG CAC AAG CGG UGG AGC AUG UGG UUU AAU UCG AAG CAA CGC GAA GAA CCU UAC CAA AUC UUG AC 3′
cDNA_16SB	DNA	5′ ACA TGC TCC ACC GCT TGT GCG GGT CCC CGT CA A TTC C 3′
FAM_R	RNA	5′ [FAM]AGC GUG ACU GAC UG 3′
DNA CA4	DNA	5′ AAA ACC AGT CAG TCA CGC T 3′
DNA TA4	DNA	5′ AAA ATC AGT CAG TCA CGC T 3′
DNA GA4	DNA	5′ AAA AGC AGT CAG TCA CGC T 3′
DNA A5	DNA	5′ AAA AAC AGT CAG TCA CGC T 3′
DNA Conly	DNA	5′ CCA GTC AGT CAC GCT 3′
DNA CA1	DNA	5′ ACC AGT CAG TCA CGC T 3′
DNA scrambled	DNA	5′ AAA ACC CGA TCG ACT CGA T 3′
RNA_18	RNA	5′ [FAM]ACA AGA UCA GUC UUU UUU 3′
RNA_15	RNA	5′ [FAM]AGC GUG ACU GAC UGG 3′
RNA_13	RNA	5′ [FAM]AAA AAC CAU AUG G 3′
cDNA_RNA15	DNA	5′ CCA CCT GTC AGT CAC GCT 3′
cDNA_AAA	DNA	5′ AAA CCC GTC AAT TCC TTT GAG TTT CAA CCT TGC GC 3′
